# Let Him Get Well

**Published:** 1899-06

**Authors:** 


### Let Him Get Well.

Dr. W. W. Keen, of Philadelphia, in discussing appendicitis at the Denver meeting of the A. M. A., concluded his remarks as follows: "I protest against the use of opium, except in rare cases, as it has a tendency to mask the symptoms of the disease and leads the patient to the grave. I protest against the argument of Dr. Niles, that every case ought to be operated upon and the appendix is never to be left. Out of 300 post-mortems on as many bodies, it was found that 100 of the individuals had had appendicitis at some time in their lives, and had all recovered from the disease. I challenge the assertion that through surgical operations all but 2 per cent. of cases can be saved. I challenge any operator in the room to take 100 well persons and operate upon them without killing more than 2 per cent. We all fail, gentlemen. I do not know why, but we all fail. I do not believe in operating on all cases of appendicitis. I'd rather have a live man with an appendix than a dead one without one. (Applause.) I do not believe with the witty Frenchman that no case is complete without a post-mortem. (Laughter.) If the patient is no worse after forty-eight hours of observation, let him alone; let him get well."—*Western Medical Review.*

